# Effect of tenofovir on renal function in patients with chronic hepatitis B

**DOI:** 10.1097/MD.0000000000009756

**Published:** 2018-02-16

**Authors:** Woo Jin Jung, Jae Young Jang, Won Young Park, Soung Won Jeong, Hee Jeong Lee, Sang Joon Park, Sae Hwan Lee, Sang Gyune Kim, Sang-Woo Cha, Young Seok Kim, Young Deok Cho, Hong Soo Kim, Boo Sung Kim, Suyeon Park, Baigal Baymbajav

**Affiliations:** a Institute for Digestive Research, Digestive Disease Center, Department of Internal Medicine, College of Medicine, Soonchunhyang University, Seoul; b Department of Internal Medicine, College of Medicine, Soonchunhyang University, Cheonan; c Department of Internal Medicine, College of Medicine, Soonchunhyang University, Bucheon; d Biostatistical Consulting Unit, Soonchunhyang University, Seoul, Korea; e UB Songdo Hospital, Ulaanbaatar, Mongolia.

**Keywords:** chronic hepatitis B, renal insufficiency, tenofovir

## Abstract

Tenofovir disoproxil fumarate (TDF) is widely used to treat patients with hepatitis B virus (HBV) infection. We investigated the effect of TDF on renal insufficiency in patients with chronic hepatitis B (CHB).

A consecutive cohort analysis was applied to CHB patients taking prescribed TDF from January 2012 to May 2016 at Soonchunhyang University Seoul Hospital. Alterations over time in corrected calcium, phosphate, creatinine, and estimated glomerular filtration rate (eGFR) were analyzed using the generalized estimating equation method. The percentage increase in creatinine from baseline to the maximum creatinine level (delta creatinine) was compared according to the underlying disease using the Mann–Whitney *U* test. Cox proportional hazard regression model was used to determine risk factors associated with renal insufficiency.

The baseline creatinine, eGFR, corrected calcium, and phosphate levels were 0.72 ± 0.01 mg/dL (mean ± SD), 106.37 ± 1.06 mL/min/1.73 m^2^, 8.82 ± 0.04 mg/dL, and 3.42 ± 0.05 mg/dL, respectively. The creatinine level had increased significantly at 12, 24, 48, 72, and 96 weeks, while the eGFR level had decreased significantly at these 5 time points. Multivariate analysis confirmed that age ≥60 years and the baseline bilirubin level were independently associated with the risk of renal insufficiency. Delta creatinine was significantly higher in patients with diabetes mellitus (DM) than in patients without DM.

Renal function was decreased from baseline in CHB patients receiving TDF therapy, which indicates that the renal function of patients undergoing treatment with TDF should be monitored regularly. Old age, DM, and serum bilirubin were risk factors for the development of renal insufficiency in CHB patients receiving TDF therapy.

## 1 Introduction

Hepatitis B virus (HBV) infection is a serious health problem in the Korean population. Positivity for hepatitis B surface antigen accounts for 70% cases of chronic hepatitis and cirrhosis, and 65% to 75% of primary liver cancer cases.^[1]^ The advances in antiviral therapy have significantly improved the prognosis of HBV infection.^[2]^ Tenofovir disoproxil fumarate (TDF) is a bioavailable prodrug of tenofovir, which is a potent nucleotide analog reverse transcriptase inhibitor with activity against human immunodeficiency virus (HIV) and HBV.^[3]^ TDF is eliminated by renal clearance, largely via glomerular filtration, with 20% to 30% being actively transported into the renal proximal tubule cells.^[4]^ TDF-associated nephrotoxicity primarily results in proximal tubular injury. Severe acute tubular necrosis was seen in 33 (77%) of 43 biopsy-proven cases of TDF nephrotoxicity.^[5]^

The tubular damage leads to defective proximal tubular secretion and reabsorption of several substances including phosphate, resulting in reduced serum phosphate levels, elevated phosphate levels in the urine and elevated serum levels of creatinine.^[6]^ A systemic review and meta-analysis of 17 studies involving HIV patients concluded that TDF exerted a small but significant effect on the loss of kidney function.^[7]^ Several case reports of HIV patients with TDF have described renal tubular dysfunction causing Fanconi syndrome.^[8–10]^ Studies of renal safety in HBV patients receiving TDF monotherapy found no evidence of compromised renal function or renal tubular dysfunction.^[11,12]^ However, conflicting data about the renal safety have been reported. Some case reports on chronic hepatitis B (CHB) patients described that Fanconi syndrome and nephrotic syndrome developed after TDF exposure.^[13,14]^ Risk factors for the renal insufficiency in CHB patients receiving TDF are not well established.

We investigated the effect of and risk factors for renal insufficiency for treatment with TDF over different periods in CHB patients.

## 2 Method

### 2.1 Study population

A consecutive cohort analysis was applied to 315 CHB patients taking prescribed TDF from January 2012 to May 2016 at Soonchunhyang University Seoul Hospital. The institutional review board of the hospital approved the study protocol. Analyses were further limited to 110 patients who had been taking TDF for at least 48 weeks, no past or concurrent use of acyclic nucleotide analogues or other nephrotoxic drugs, no previous renal insufficiency (creatinine ≤ 1.5 mg/dL, estimated glomerular filtration rate [eGFR] ≥60 mL/min), and measurements of corrected calcium, phosphate, creatinine, and eGFR levels during therapy. The patients’ follow-up period ranged from 48 to 171 weeks (median 103).

### 2.2 Data collection

The following demographic, clinical, and laboratory data reflecting potentially predictive variables of interest were collected: age, sex, body mass index (BMI), duration of therapy, baseline creatinine, eGFR, calcium, phosphorus, uric acid, sodium, potassium, albumin, aspartate aminotransferase, alanine transaminase, and bilirubin levels, HBV DNA, underlying disease such as hepatitis C virus (HCV) or HIV coinfection, liver cirrhosis (LC), hypertension (HTN), and DM. BMI was calculated as weight in kilograms divided by height in meters squared. The duration of therapy was calculated as the time between the starting date and the last date of treatment with TDF, defined as the last dispensation of TDF or the date of the end of the study. Information about underlying disease was collected from medical records.

### 2.3 Estimated glomerular filtration rate

eGFR was calculated using the Chronic Kidney Disease Epidemiology Collaboration (CKD-EPI) equation, which is recommended for routine clinical use instead of the Modification of Diet in Renal Disease (MDRD) equation because the CKD-EPI equation is more accurate than the MDRD equation across a wide variety of populations and clinical conditions.^[15,16]^

### 2.4 Statistical analyses

To investigate the effect of TDF on renal function during the treatment period, alterations over time in the corrected calcium, phosphate, creatinine, and eGFR levels were analyzed using the generalized estimating equation method. The percentage increase in creatinine from baseline to the maximum creatinine level (delta creatinine) was compared according to underlying conditions (HTN, DM, BMI ≥25 kg/m^2^, and age ≥60 years) using the Mann–Whitney *U* test. Renal insufficiency was defined as a creatinine level of ≥0.3 mg/dL or a ≥1.5-fold increase from the baseline creatinine level, in accordance with the acute kidney injury definition in Kidney Disease Improving Global Guidelines.^[17]^ Recovered acute kindey injury until the end of the follow-up was not considered as renal insufficiency. Hypophosphatemia was defined as a phosphorus level of ≥2.8 mg/dL at baseline but <2.8 mg/dL during the treatment period. Cumulative probabilities of renal insufficiency through TDF treatment were estimated using Kaplan–Meier analysis. The Cox proportional hazard regression model was used to identify the univariate and multivariate risk factors for renal insufficiency. The logistic regression models were used to determine risk factors associated with hypophosphatemia. The selection threshold for entering variables in the multivariate analysis was a *P* value of <.05 in the univariate analysis. Data were analyzed using SPSS (version 22.0).

## 3 Result

### 3.1 Study population

**Figure 1:**
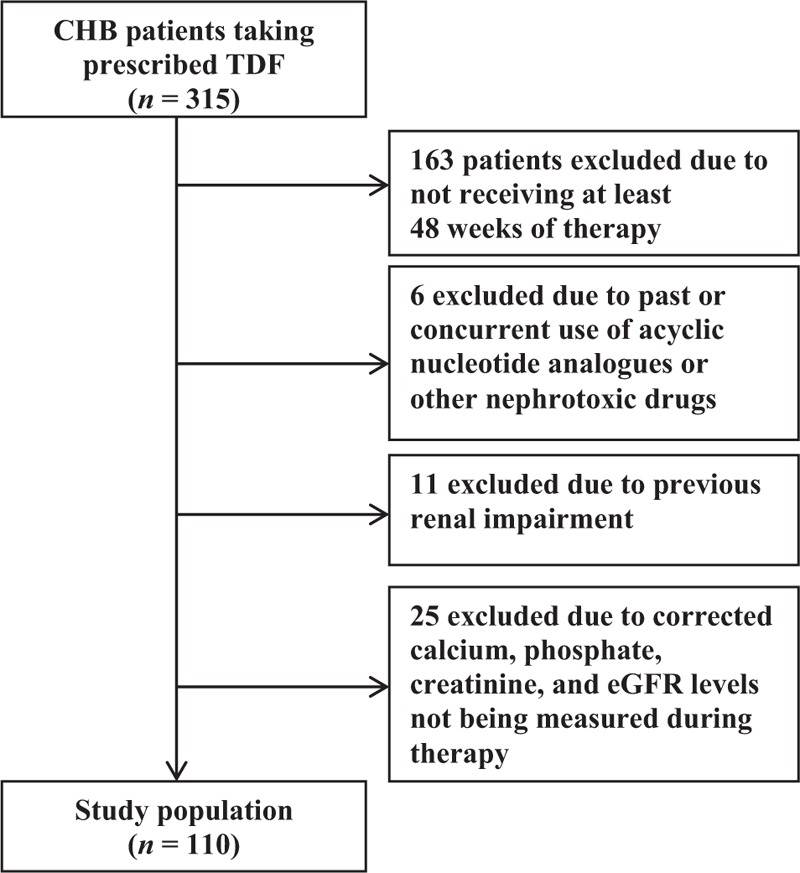
Flow chart of the study population. After excluding 205 of the 315 initially enrolled patients, the study population comprised 110 patients. CHB = chronic hepatitis B, eGFR = estimated glomerular filtration rate, TDF = tenofovir disoproxil fumarate.

During the recruitment period from January 2012 to May 2016, 315 patients were assessed for eligibility, of whom 205 were excluded: 163 due to not receiving at least 48 weeks of therapy with TDF, 6 due to past or concurrent use of acyclic nucleotide analogs or other nephrotoxic drugs, 11 due to previous renal insufficiency, and 25 due to the corrected calcium, phosphate, creatinine, and eGFR levels not being measured during therapy (Fig. 1). The baseline characteristics of the 110 patients included in the analysis are listed in Table 1. Their age was 51.3 ± 11.3 years (mean ± SD), and 65.5% of the patients were males. The duration of therapy was 102.8 ± 37.2 weeks. In terms of underlying diseases, there were 40 (36.4%), 15 (13.6%), and 15 (13.6%) patients with LC, HTN, and DM, respectively. None of the included patients had HCV or HIV coinfection. The baseline creatinine, eGFR, corrected calcium, and phosphate levels were 0.72 ± 0.01 mg/dL, 106.37 ± 1.06 mL/min/1.73 m^2^, 8.82 ± 0.04 mg/dL, and 3.42 ± 0.05 mg/dL, respectively. The albumin and bilirubin levels were 3.8 ± 0.7 g/dL and 1.2 ± 1.4 mg/dL, respectively. The HBV DNA log IU value was 5.7 ± 2.2, and 89 of the 110 patients (80.9%) had HBV DNA >2000 IU at baseline.

**Table 1:**
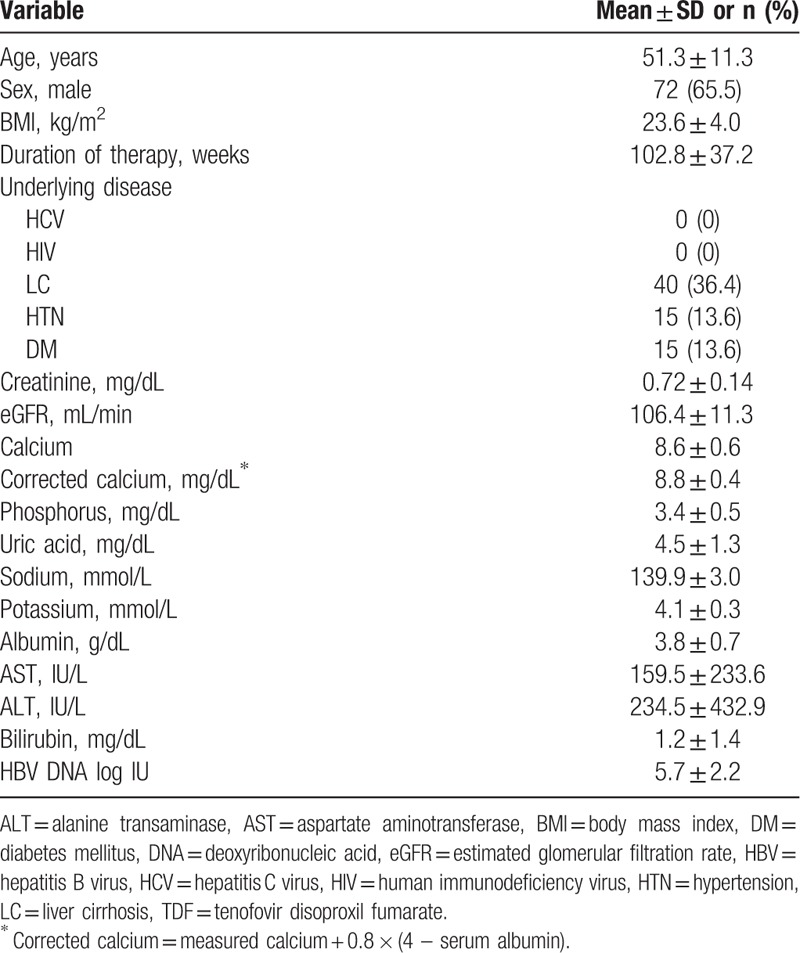
Baseline characteristics of patients treated with TDF.

### 3.2 Mean changes in creatinine, eGFR, corrected calcium, and phosphorus levels

**Figure 2:**
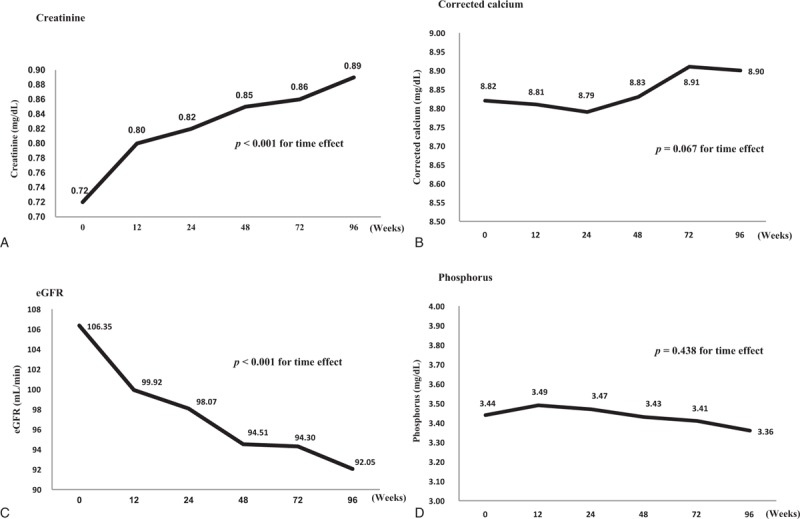
Changes in creatinine, eGFR, corrected calcium, and phosphorus levels over time using the generalized estimating equation method. The cumulative mean changes in creatinine (A) and eGFR (B) were significantly related to the TDF exposure period. The mean corrected calcium (C) and phosphate (D) levels during therapy did not differ significantly from the baseline values. eGFR = estimated glomerular filtration rate, TDF = tenofovir disoproxil fumarate.

The cumulative mean changes in the creatinine, eGFR, corrected calcium, and phosphorus levels were calculated after 12, 24, 48, 72, and 96 weeks of exposure to TDF (Table 2). The mean creatinine level had increased significantly at 12 (+0.08, *P* <.001), 24 (+0.10, *P* <.001), 48 (+0.13, *P* <.001), 72 (+0.14, *P* <.001), and 96 weeks (+0.17, *P* <.001). The cumulative mean change in creatinine was significantly related to the TDF exposure period (*P* <.001). The eGFR had decreased significantly at 12 (−6.43, *P* <.001), 24 (−8.28, *P* <.001), 48 (−11.84, *P* <.001), 72 (−12.06, *P* <.001), and 96 weeks (−14.30, *P* <.001) (Table 2, Fig. 2). There was a significant time effect in the cumulative mean change in eGFR level (*P* <.001). The mean corrected calcium and phosphate levels during therapy did not differ significantly from the baseline levels.

**Table 2:**
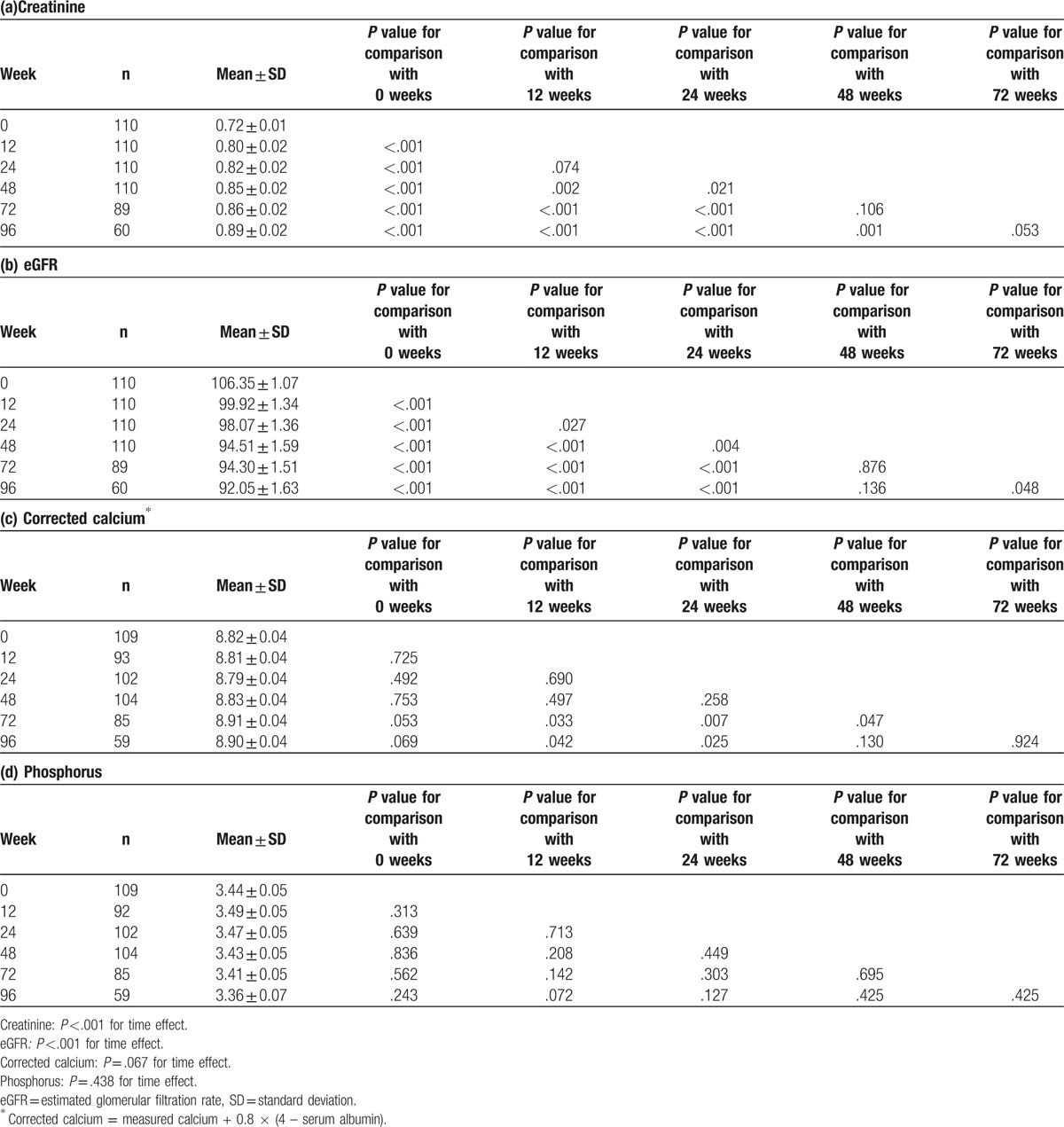
Differences in creatinine, eGFR, corrected calcium, and phosphorus levels from baseline using the generalized estimating equation method.

### 3.3 Difference in delta creatinine according to underlying conditions

**Figure 3:**
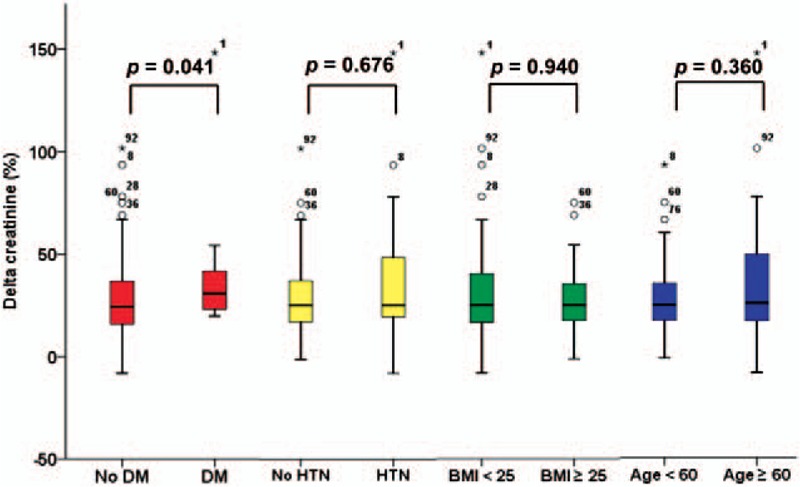
Differences in delta creatinine according to the underlying disease. Delta creatinine was significantly higher in patients with DM than in patients without DM (40.23 vs 27.86, *P* = .041), but it did not differ significantly between patients with or without HTN, age ≥60 or <60 years, and BMI ≥25 or <25 kg/m^2^. BMI = body mass index, DM = diabetes mellitus, HTN = hypertension.

The overall delta creatinine value in CHB patients receiving TDF was 29.5 ± 22.2%: it was 39.8 ± 40.2%, 40.2 ± 31.6%, 27.4 ± 17.4%, and 38.3 ± 34.5% in patients with HTN, DM, age ≥60 years, and BMI ≥ 25 kg/m^2^, respectively. Delta creatinine was significantly higher in patients with DM than in patients without DM (40.23% vs 27.86%, *P* = .041, Fig. 3), but it did not differ between patients with or without HTN, age ≥60 or <60 years, and BMI ≥25 or <25 kg/m^2^.

### 3.4 Risk factors for developing renal insufficiency and hypophosphatemia

**Figure 4:**
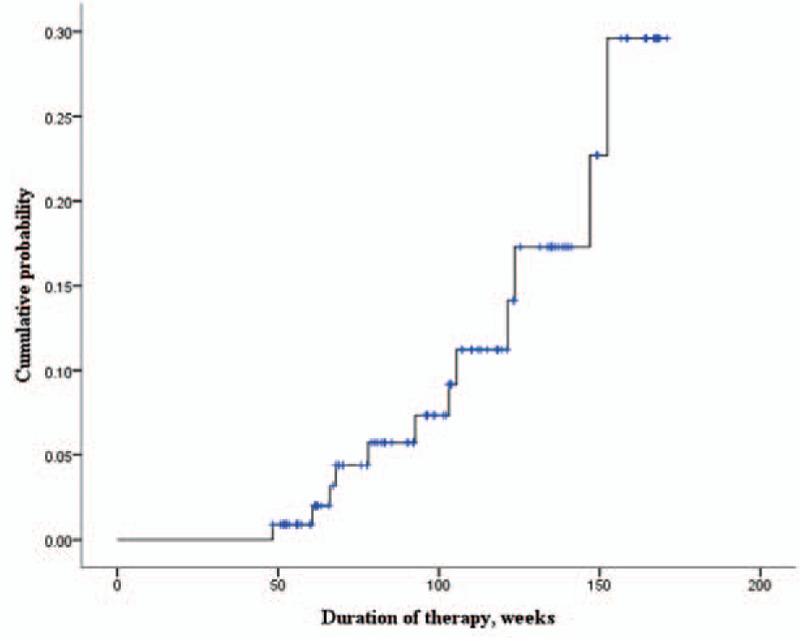
Cumulative probability of renal insufficiency through TDF treatment. TDF = tenofovir disoproxil fumarate.

The cumulative probability of renal insufficiency through TDF treatment is shown in Figure 4. We dichotomized the 110 patients receiving TDF according to whether (n = 12, 10.9%) or not (n = 98, 89.1%) they developed renal insufficiency (Table 3). Age ≥60 years and the baseline phosphorus, albumin, and bilirubin levels showed significant associations with renal insufficiency. A multivariate analysis adjusting for these variables confirmed independent associations with the risk of renal insufficiency for age ≥60 years (odds ratio [OR] = 3.866, 95% confidence interval [CI] = 1.192–12.538) and baseline bilirubin level (OR = 1.324, 95% CI = 1.055–1.661), while BMI ≥25 kg/m^2^, the baseline creatinine, eGFR, phosphorus, and albumin levels, HBV DNA >2000 IU, and underlying diseases such as HTN and DM were not significantly associated with the development of renal insufficiency in CHB patients receiving TDF. Hypophosphatemia occurred during the treatment period in 18 of the 110 patients (16.9%). In univariate logistic analysis, patients with HTN had a tendency to develop hypophosphatemia (*P* = .066, Table 4).

**Table 3:**
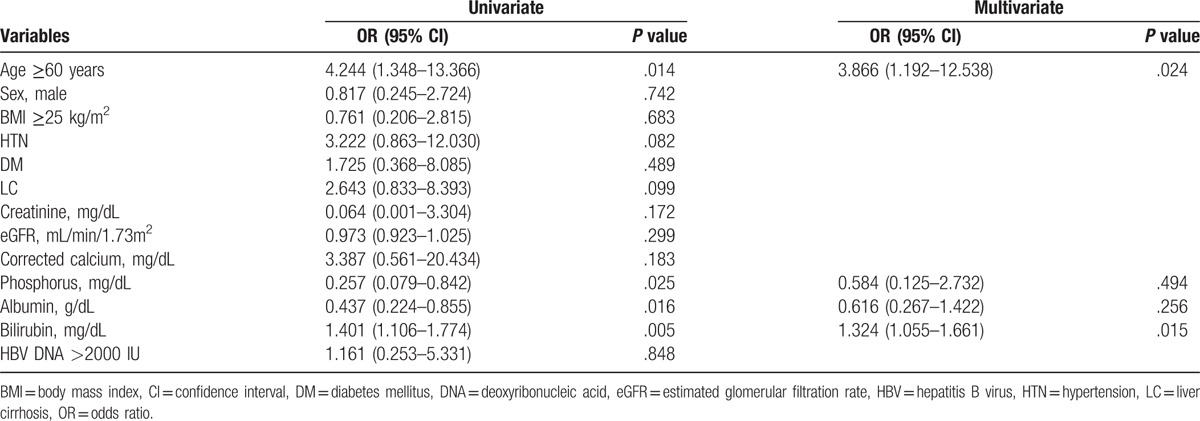
Results from the Cox proportional hazard regression model of risk factors for renal insufficiency.

**Table 4:**
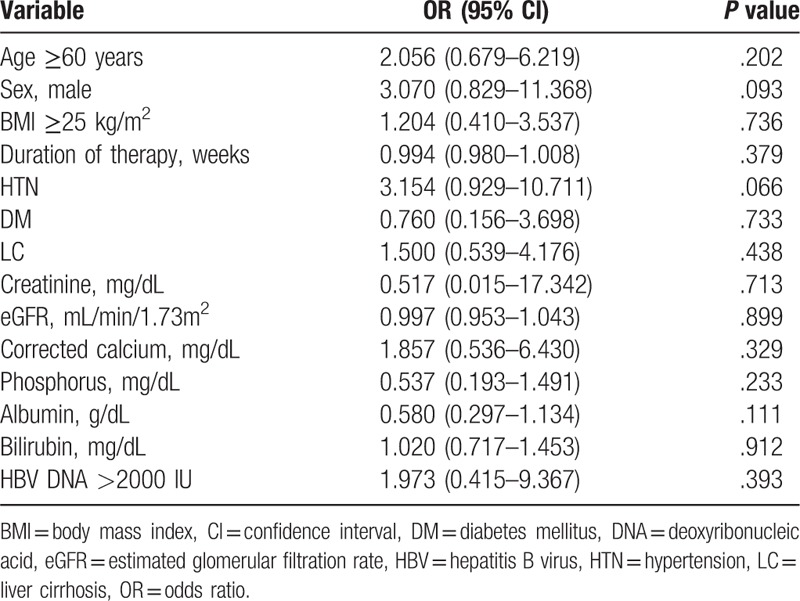
Results from the univariate logistic regression model of risk factors for hypophosphatemia.

## 4 Discussion

TDF is one of the most potent antiviral agents against HBV and HIV. However, its potential effects on renal insufficiency are still being questioned. Randomized controlled clinical trials involving HIV patients, which have generally excluded patients with a history of renal disease or renal insufficiency, have indicated a similar incidence of renal adverse events in TDF-treated patients, comparable to the rates observed in placebos.^[18,19]^ Another studies in Korean CHB patients showed very low incidence (2.7%, 2.9%) of renal adverse event.^[20,21]^ A matched case–cohort study of CHB patients found no significant difference in the proportion of patients reclassified to a more severe renal classification or in the proportion of patients exhibiting a decrease in eGFR of ≥20% in those exposed to TDF versus control.^[22]^ On the other hand, some studies have found TDF to be associated with a decrease in eGFR. In a study of 273 CHB patients receiving TDF for >6 months, the eGFR level at 24 months was lower than that at baseline (–3.99 mL/min/1.73 m^2^).^[23]^ Another study of 50 CHB patients receiving TDF for >12 months found that the median eGFR level at the end of the follow-up was lower than at baseline (–6 mL/min/1.73 m^2^).^[24]^ A multivariate analysis of 619 CHB patients receiving either entecavir (n = 547) or TDF (n = 72) revealed that increasing age, male sex, HTN, abnormal baseline eGFR, cirrhosis, and TDF therapy were independent factors associated with a higher probability of acute kidney injury.^[25]^ However, these studies had limitations of small populations, a short duration of therapy, or using eGFR as calculated by the MDRD equation. In the present study, we have clearly showed that long-term TDF therapy caused a decline in eGFR in CHB patients. Data from our investigations demonstrate that TDF therapy for >48 weeks influenced renal function during the treatment period, despite including patients without renal insufficiency and other nephrotoxic agents.

Several predisposing factors were defined for TDF-induced renal insufficiency. In a cohort study involving 10,343 HIV patients, elevated serum creatinine, concomitant use of nephrotoxic medications, increased age, lower weight, and lower cluster of differentiation (CD)4 cell count were baseline risk factors for the development of TDF-induced renal insufficiency.^[26]^ In a retrospective study of 172 HIV patients, only baseline creatinine and baseline creatinine clearance were associated with an increase in serum creatinine of at least 1.5-fold relative to baseline.^[27]^ No significant relationship was identified with other potential confounding variables, including the use of nephrotoxic medication, increased age, or baseline CD4 cell count. A prospective observational study of 354 HIV patients receiving TDF showed that patients with TDF nephrotoxicity were older and more frequently males, HCV co-infected, in Centers for Disease Control and Prevention stage C, and had a CD4 cell count that was significantly lower than those without nephrotoxicity.^[28]^ In a study involving 135 HBV and HIV/HBV-infected patients receiving TDF, age, non-African origin, baseline eGFR, and a baseline HBV DNA level of >2000 IU/mL were associated with a decrease in the eGFR level.^[24]^

In the present study, delta creatinine differed significantly between those with and without DM. Our multivariate analysis of risk factors for renal insufficiency found that old age and the baseline bilirubin level were significantly associated with the development of renal insufficiency. In contrast to the findings of previous studies, baseline creatinine, low body weight, and HBV DNA >2000 IU/mL were not associated with renal insufficiency in the present study. These discrepant findings might have been due to our study population including relatively stable patients with normal renal function, compared with other studies.

It is uncertain why higher total bilirubin level was associated with the development of renal insufficiency in our study. In a study of 4178 CHB patients receiving antiviral agents, patients exhibiting the rapid progression of chronic kidney disease showed lower serum albumin, higher total bilirubin, and prolonged prothrombin time compared to patients with stable renal function.^[29]^ A reasonable explanation is that patients with hepatic dysfunction are prone to renal insufficiency.

Hypophosphatemia is a possible complication in patients with TDF. There have been several case reports of proximal renal tubular dysfunction and the Fanconi syndrome in TDF-treated patients, which lead to excessive urinary excretion of solutes handled by the proximal tubule, such as phosphate, glucose, and bicarbonate.^[30–32]^ Tien et al^[33]^ reported on a prospective cross-sectional study involving 146 Asian–American CHB patients who received no treatment (n = 60), or treatment with TDF (n = 42) or entecavir (n = 44). Although the mean serum phosphate levels were similar in the TDF and entecavir groups, the proportion of patients with hypophosphatemia (phosphorus <2.8 mg/dL) was significantly higher in the TDF group than in the entecavir group (48% vs 12%, *P* = .005). However, in a double-blind, placebo-controlled study of 552 HIV patients, grade 1 or 2 hypophosphatemia occurred in 12% and 7% of TDF-treated and placebo-treated patients, respectively, with no significant intergroup difference (*P* >.05).^[34]^ In a study of 423 HBV patients (TDF-initiated and TDF-switched), only 7 patients developed transient hypophosphatemia and no patients had to change their TDF treatment.^[23]^ In the present study, 18 of 110 patients (16.9%) developed hypophosphatemia during the treatment period. Patients with HTN exhibited a tendency to develop hypophosphatemia, while the phosphorus level did not differ significantly from the baseline during the treatment period.

The guideline of the European Association for the Study of the Liver (EASL) states that all patients starting TDF should be tested for serum creatinine levels before treatment, and the baseline renal risk should be determined, which includes assessing one or more of the following factors: decompensated cirrhosis, creatinine clearance <60 mL/min, poorly controlled HTN, proteinuria, uncontrolled DM, active glomerulonephritis, concomitant nephrotoxic drugs, and solid-organ transplantation.^[35]^ The 2016 American Association for the Study of Liver Diseases guideline recommends that the serum creatinine, phosphorus, urine glucose, and urine protein levels should be measured in persons taking TDF both before treatment initiation and periodically during the treatment (e.g., at least annually, and more frequently if there was pre-existing renal dysfunction or a high risk of renal dysfunction).^[36]^ According to the 2016 Asian-Pacific Association for the Study of the Liver guideline, due to the potential nephrotoxicity of TDF, serum creatinine and phosphate levels should be monitored every 3 months during TDF therapy.^[37]^ However, these guidelines (with the exception of the EASL one) do not mention the risk factors for renal insufficiency in patients with TDF. Our study found that even in CHB patients receiving TDF with normal renal function and no nephrotoxic medication, the serum creatinine level was significantly elevated from the baseline during the treatment period. Patients with risk factors such as DM, old age, and high serum bilirubin should be cautioned about the possibility of renal insufficiency when initiating TDF.

The present study has some limitations. First, it was a retrospective single center study. Second, we could not directly compare the patients taking TDF with the patients not taking TDF. Finally, study population was relatively small.

## 5 Conclusion

Renal function was decreased relative to baseline in CHB patients receiving TDF therapy, which indicates that the renal function should be monitored regularly in patients undergoing treatment with TDF. Old age, DM, and the serum bilirubin level were risk factors for the development of renal insufficiency in CHB patients receiving TDF therapy.
